# Primary exploration of host–microorganism interaction and enteritis treatment with an embedded membrane microfluidic chip of the human intestinal–vascular microsystem

**DOI:** 10.3389/fbioe.2022.1035647

**Published:** 2022-12-06

**Authors:** Wei Zhao, Yuhan Yao, Tong Zhang, Huijun Lu, Xinlian Zhang, Linlin Zhao, Xi Chen, Jinhui Zhu, Guodong Sui, Wang Zhao

**Affiliations:** ^1^ Shanghai Key Laboratory of Atmospheric Particle Pollution Prevention (LAP3), Department of Environmental Science and Engineering, Fudan University, Shanghai, China; ^2^ Shanghai Changhai Hospital Department of Gastroenterology, Shanghai, China

**Keywords:** gut-on-a-chip, embedded membrane chip, ESBL, enteritis, treatment

## Abstract

Intestinal flora plays a crucial role in the host’s intestinal health. Imbalances in the intestinal flora, when accompanied by inflammation, affect the host’s intestinal barrier function. Understanding it requires studying how living cells and tissues work in the context of living organs, but it is difficult to form the three-dimensional microstructure intestinal–vascular system by monolayer cell or co-culture cell models, and animal models are costly and slow. The use of microfluidic-based organ chips is a fast, simple, and high-throughput method that not only solves the affinity problem of animal models but the lack of microstructure problem of monolayer cells. In this study, we designed an embedded membrane chip to generate an *in vitro* gut-on-a-chip model. Human umbilical vein endothelial cells and Caco-2 were cultured in the upper and lower layers of the culture chambers in the microfluidic chip, respectively. The human peripheral blood mononuclear cells were infused into the capillary side at a constant rate using an external pump to simulate the *in vitro* immune system and the shear stress of blood *in vivo*. The model exhibited intestine morphology and function after only 5 days of culture, which is significantly less than the 21 days required for static culture in the Transwell^®^ chamber. Furthermore, it was observed that drug-resistant bacteria triggered barrier function impairment and inflammation, resulting in enteritis, whereas probiotics (*Lactobacillus rhamnosus* GG) improved only partially. The use of Amikacin for enteritis is effective, whereas other antibiotic therapies do not work, which are consistent with clinical test results. This model may be used to explore intestinal ecology, host and intestinal flora interactions, and medication assessment.

## Introduction

The intestine is the longest organ of the human body’s digestive tract and plays an important role in the digestion, absorption, and secretion functions (Atarashi et al., 2017). The intestinal microbiota found in human and other animal digestive tracts comprise a complex community of microbes that coexist in host mucosal epithelial cells, which are closely tied to blood vessels (Garrett et al., 2010) and the immune system (Thomas et al., 2017). Intestinal microbiota plays an important role in maintaining intestinal homeostasis (Tremaroli and Backhed, 2012). Consequently, constructing a small intestine model is crucial for the study of the interactions between gut microorganisms and their hosts.

Researchers initially only cultured one type of cell (Caco-2 or HT-29). Bilayer cell static culture methods were then gradually utilized to produce the small intestinal barrier model (Vizoso Pinto et al., 2009; Calatayud et al., 2019), which is widely used for drug development (Per Arturssona’* et al., 1996; S et al., 1990). Although an epithelial barrier is established in these procedures, the cells lack the columnar structure shown *in vivo* to better mimic the differentiated organ-specific features of the human gut. The first intestinal organoid was constructed in 2009 (Sato et al., 2009), signaling the beginning of a new era of *in vitro* organ models. The model utilizes extracellular matrix (ECM) to offer a 3D structure and a growth microenvironment more closely resembling the *in vivo* microenvironment, allowing stem cells to differentiate into structures that resemble intestinal cavities. Since then, researchers have built an increasing number of organoids (Jason et al., 2009; Jung et al., 2011; Sato et al., 2011; Chua et al., 2014; DeWard et al., 2014; Karthaus et al., 2014; Ren et al., 2014; Bartfeld et al., 2015; Boj et al., 2015; Huch et al., 2015; Kessler et al., 2015; Maimets et al., 2016; Turco et al., 2017; Sachs et al., 2018), but intestinal organoids still have drawbacks, such as a long build time, difficulty removing dead cells.

Because of the combination of microsystems engineering and cell biology, chips for cell culture have been able to precisely control the shape, location, and function of cells in highly structured cell culture scaffolds (Kim et al., 2012). Some gut-on-a-chip system simulate external mechanical stress, pipeline shearing force, and other *in vivo* environmental simulation characteristics, thus shortening the culture period from 21 days to only 5 days. Simultaneously, it possesses the structure, functions, secretes mucus and enzymes of the small intestine. In addition, numerous other organs-on-a-chip have been created (Huh et al., 2010; Wei et al., 2011; Huh et al., 2012; Snouber et al., 2012; Agarwal et al., 2013; Maschmeyer et al., 2015; Jie et al., 2016; Li et al., 2016; Nierode et al., 2016; Oleaga et al., 2016; Du et al., 2017; Delalat et al., 2018; Jain et al., 2018; Deng et al., 2019; Theobald et al., 2019; Zhang et al., 2020). Today, organ-on-a-chip increasingly resembles the real organ, take gut-on-a-chip as an example, some models simulate probiotics or flora colonization (Kim et al., 2012; Jing et al., 2020), whereas others simulate the anaerobic environment in the intestine (Jalili-Firoozinezhad et al., 2019).

Drug mechanisms and drug screenings have also been studied (Maschmeyer et al., 2015; Choe et al., 2017; Kyall Pocock and Vaskor, 2017; Li et al., 2017). Aside from the standard small intestine model, scientists have attempted to model human intestinal inflammation *in vitro* using organ-on-a-chip technology (Jing et al., 2020; Nikolaev et al., 2020). Some researchers have used a model for intestinal inflammatory responses caused by *Escherichia coli* (*E. coli*). However, the use of non-pathogenic bacteria that settle in the digestive system to mimic enteritis is not the best choice. This study utilized *E. coli* that produce extended-spectrum β-lactamase enzymes (ESBL-EC) isolated from the intestinal tract to imitate enteritis caused by antibiotic-induced flora imbalance. ESBL-EC is not sensitive to β-lactam antibiotics such as penicillin, cephalosporins, aztreonam, and other antibiotics; it may cause faster plasmid transmission of drug resistance genes, causing significant clinical problems. Presently, drug-resistant *E. coli* is prevalent worldwide. The types and breadth of drug resistance in *E. coli* have increased, as well as the characteristics of cross-resistance and multi-resistance (HAN and Han, 1996; Anucha Apisarnthanarak et al., 2008). Antibiotic use may cause dysbacteriosis, lasting more than a year after treatment is stopped. Creating a model of drug-resistant bacteria enteritis is crucial to comprehending the critically important effects of drug-resistant bacteria on the intestine.

Lots of organ-on-a-chip models use microfluidic chips with similar structures (Kim et al., 2012; Kim and Ingber, 2013; Kim et al., 2016; Villenave et al., 2017; Shin et al., 2019; Ambrosini et al., 2020; Nelson et al., 2021), but the thin, porous polydimethylsiloxane (PDMS) layer in the middle of the chip is difficult to manufacture. Consequently, many researchers prefer polycarbonate (PC) film to PDMS film, but we found that PC film and PDMS adhesion are not strong enough compared with PDMS film. In order to solve this problem, a unique human intestinal microfluidic chip was constructed that effectively avoided the restrictions above in this study. First, the embedded membrane microfluidic chip’s architecture increases the chip’s durability. Compared with the thin PDMS membrane, the PC membrane has superior biocompatibility. The sealing channel design ensures that the PC film and PDMS are firmly embedded. Second, compared with the earlier gut-on-a-chip (Kim et al., 2016), which only used Caco-2 to simulate the intestinal, the gut-on-a-chip system constructed in this study contains the intestinal cavity, the vascular cavity (containing PBMC), and probiotics colonized in the intestinal cavity. The shear forces are simulated using syringe pumps. This system is closer to the real situation of intestinal tract *in vivo*. Finally, we created the inflammatory model of intestinal injury produced by clinically isolated drug-resistant bacteria and tested the therapeutic effects of *Lactobacillus rhamnosus* GG (LGG) and antibiotics.

## Experimental section

### Materials and reagents

PDMS (RTV615) was purchased from Momentive (Waterford, NY, United States). SU-8 2075 was purchased from MicroChem (Newton, MA, United States). Polycarbonate membranes with pore sizes of 8 μm was purchased from Whatman (110414; Maidstone, United Kingdom). *L. rhamnosus* GG (LGG) was obtained from American Type Culture Collection (ATCC 53103; Manassas, VA, United States), which was originally isolated from the human gut (Saxelin, 2009). *E. coli* with extended-spectrum β-Lactamase (ESBL-EC) was provided by the Department of Clinical Laboratory, Shanghai East Hospital. Antibodies were bought from Santa Cruz Biotechnology (United States) and Novoprotein (Shanghai, China).

### Fabrication and assemblage of the microfluidic device

The microfluidic devices of gut-on-a-chip used in this study include the sealing channel, PC porous membranes, and cell microculture chambers. Briefly, in order to bond the PC and the upper and lower cell culture chambers of the gut-on-a-chip device more firmly, PDMS pre-polymer was mixed with curing agent of 10:1 (w:w) and 8:1 (w:w), and the mixture was then cast onto two silicon wafers with 150 μm thick patterns of SU-8 2075 separately and cured at for 2 h at 80°C. The cured PDMS layer was peeled from the wafer separately. Holes of 1 mm diameter were drilled through the cured PDMS to serve as inlets or outlets.

**FIGURE 1:**
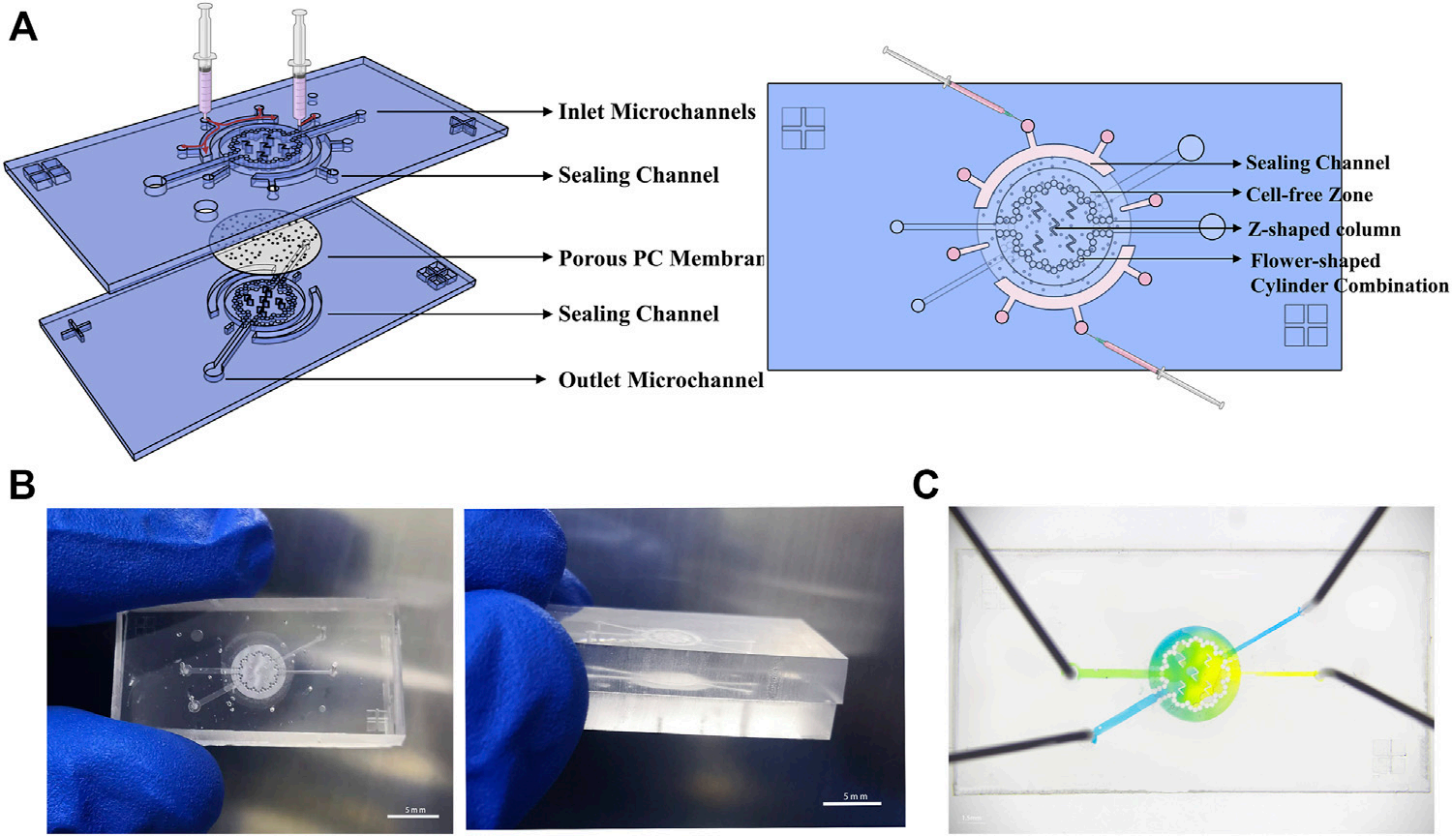
Microsystem for gut-on-a-chip. **(A)** Three-dimensional schematics of the device show structure of individual components and the sealing method. **(B)** Photographs of the chip’s physical diagram were taken from various angles. **(C)** An image of the device. The upper and bottom microchannels are filled with yellow and blue dyes, respectively.

Between two pieces of PDMS containing channel features, PC membranes with pore sizes of 8 μm served as the middle layer (Figure 1A). A membrane with a diameter of 400 μm was gently sandwiched in the sealing channel with a diameter of 500 μm (sealing tube pre-treated by plasma). The assembled chip was pre-cured at 80°c for 5 min. The membrane edge was then soaked with PDMS pre-polymer combined with a curing agent at a 9:1 (w: w) ratio and fed into the sealing channel from two inlets at a flow rate of 10 μl min^−1^. The microfluidic device was then cured at 80°C for 10 h. Then, the chip was pre-treated with 75% ethanol, autoclaved at 121°C for 30 min, and dried in an oven at 60°C.

### Cell culture

The human intestinal epithelial cells Caco-2 from the National Collection of Authenticated Cell Cultures (NCACC) were cultured in Modified Eagle Medium (MEM, Gibco) supplemented with 15% (w/v) of fetal bovine serum (FBS, Gibco). The human umbilical vein endothelial cells (HUVECs) were provided by Shaorui Ke from the Henan University of Chinese Medicine and cultured in Endothelial Cell Medium (Scciencell Shanghai, China) containing 5% (w/v) of fetal bovine serum and 1% endothelial cell growth supplements (ECGS). Peripheral blood mononuclear cells (PBMC, Sciencell Shanghai, China) were cultured in RPMI 1640 medium (RPMI 1640, Gibco) containing 10% (w/v) of FBS. Penicillin (100 units/mL, Gibco) and streptomycin (100 mg/ml, Gibco) were added to all mediums mentioned above. All cells were cultured in a cell incubator with 5% CO_2_ at 37°C. Antibiotics were removed from the culture medium when gut chip co-culture with living microbes.

### Establishing the gut-on-a-chip

Both chambers of the chip were coated with type I collagen (30 μg/ml; Gibco) and Matrigel (100 μg/ml; BD Biosciences, Bedford, MA, United States) in serum-free MEM, which was injected into the microchannels and incubated for 1 h before cell plating. Caco-2 cells (1 × 10^5^ cell/cm^2^) were stained with 5 mM green cell-tracker (CMFDA Dye, Invitrogen) were seeded in the lower microculture chamber, and the chip was turned upside down and incubated at 37°C allowing the seeded intestinal epithelial cells to grow on the membrane surface, Caco-2 static culture for 1 day, then continuously perfused into Caco-2 chambers at 60 μl/h using a multi-channel injection pump (LSP04-1A, LONGER Halma, England). After 2 days, HUVECs (1 × 10^5^ cells/cm^2^) stained with 5 mM red cell-tracker (CMPTX Dye, Invitrogen) were seeded into the upper microculture chamber and static culture for 1 day, then continuously perfused into all chambers at 60 μl/h using a multi-channel injection pump. After 3 days, PBMC (1 × 10^7^ cells/ml) with fresh antibiotic-free culture medium (50% HUVECs culture medium and 50% PBMC medium) was continuously perfused into the upper chamber (capillary side) at 60 μl/h using a multi-channel injection pump.

### Establishing the intestinal on the Transwell^®^ chamber

For the static model, 1 × 10^5^ cell/cm^2^ Caco-2 cells were stained with CellTracker™ Green CMFDA (5 mM), then it was seeded onto the basal of the Transwell^®^ chamber with collagen type I hydrogel and Matrigel-coated membrane (6.5 mm diameter, 8 μm pore size PC membrane), the Transwell^®^ was turned upside down and incubated at 37°C for 2 h allowing the seeded intestinal epithelial cells to grow on the membrane surface, then turn it upside down again and add fresh culture medium. After 2 days, HUVECs cells stained with CellTracker™ Red CMPTX (5 mM) were plated in the upper chambers. After 3 days, PBMC (1 × 10^7^ cells/ml) was added to the upper chamber.

### Setting up the inflammatory bowel disease model caused by ESBL-EC on the chip

The ESBL-EC was obtained from patients with pathological tissue of the human intestine. After adjusting the ESBL-EC density to 1 × 10^7^ CFU/ml, it was centrifuged at 10,000G for 5 min and stained in the antibiotics-free and serum-free MEM medium with 5 mM CMTPX or CMFDA at 37°C for 30 min. Then ESBL-EC were resuspended in antibiotic-free 10% FBS MEM medium immediately and flowed into the lumen side. At the same time, PBMC (1 × 10^7^ cells/ml) were introduced into the capillary side at 60 μl/h. After ESBL-EC was allowed to attach to the surface of the gut for 2 h under static conditions, a fresh antibiotic-free but containing PBMC culture medium was perfused continuously into capillary microchannels for 1 day. Amounts of IL-8, IL-6, IL-1β, and TNF-α were analyzed using an enzyme linked immunosorbent assay (ELISA) kit (1110802, 1110602, 1110122, 1117202; Dakewe Bio-engineering).

### Anti-inflammatory evaluation of *L. rhamnosus* GG and antibiotics

To evaluate the anti-inflammatory effect of LGG, probiotics was resuspended in a mixture of sterilized MRS Broth (1.10661.0500, MERCK, Darmstadt, Germany) and static culture at 37°C for 12 h. After cell density was adjusted to 1 × 10^7^ CFU/ml, LGG was mixed with ESBL-EC and injected into the Caco-2 channel.

For the antibiotic anti-inflammatory evaluation, 1 × 10^7^ CFU/ml ESBL-EC was added into the Caco-2 channel. Then using Ceftazidime (30 μg/ml, 60 μg/ml TCI, Shanghai, China), Amikacin (30 μg/ml, 60 μg/ml OKA, Beijing, China) or penicillin (100 units/ml, Gibco) and streptomycin (100 mg/ml, Gibco) (P-S)were added to the culture medium of the lumen layer, PBMC side keeps static. After 1 day, ELISA (mentioned above) and counting (mentioned below) were conducted.

### Morphological studies

Samples were fixed with 4% paraformaldehyde (P0099, Beyotime) for 15 min, permeabilized with 0.1% Triton X-100 (P0096, Beyotime) for 10 min, and blocked with Blocking Buffer (P0200, Beyotime) for 10 min at room temperature. To visualize tight epithelial junctions, cells were then incubated with Occludin monoclonal antibody (sc-271842, CRUZ biotechnology) at 1:100 in Immunol staining primary antibody dilution buffer (P0103, Beyotime) for 4°C overnight, washed with PBS. To visualize the protein of the intestinal epithelial polarization and specific structure, cells were then incubated with the F-actin polyclonal antibody (130935, Abcam), villi polyclonal antibody (sc-58897, CRUZ biotechnology) at 1:100 in primary antibody dilution buffer for 4°C overnight, and washed with PBS, then incubated with an Alexa Fluor 488-labeled Goat anti-Mouse IgG (H + L) Secondary Antibody (A0428, Beyotime) at a dilution of 1:500 for 2 h at room temperature. For visualize the carbohydrate of the intestinal epithelial mucus layer, Caco-2 was incubated with fluorescein isothiocyanate labeled wheat germ agglutinin (WGA, Sigma) for 30 min at 37°C, then washed with PBS. Nuclei were stained with 2-(4-Amidinophenyl)-6-indolecarbamidine dihydrochloride (DAPI, C1002, Beyotime) or Hoechst 33258 (23491-45-4, Sigma). For the acidic mucopolysaccharides within the mucus, gut-on-a-chip was stained with alcian blue (pH 2.5, Biotime) by flowing the solution into microchannela for 12 h after fixed with 4% paraformaldehyde, and then washing with PBS.As a negtive control, Caco-2 clutured in the Transwell^®^ chamber for 21 days. Images were taken under inverted fluorescence microscopy (Nikon).

### Measurement of paracellular permeability

The barrier-forming capacity of the gut-on-a-chip was evaluated by measuring the apparent permeability coefficients (*Papp*) of FITC-labeled dextran with different molecular weights (3–5, 40, and 70 kDa, Sigma) through the microfluidic chip. The FITC-dextran (1 μM) was perfused through the lumen microchannel, and the D’Hanks solution was add into the capillary microchannels and detect fluorescence intensity. The equation mentioned by Kim et al. (2012).

*Papp* = $\left(\frac{dQ}{dt}\right)\left(\frac{1}{{AC}_{0}}\right)$

Where *A* is the surface area of the chamber (cm^2^), *C*_*0*_ is the initial concentration (mM), and Q is the number of absorbed molecules (mol).

### Measurement of alkaline phosphatase activity

Human intestinal epithelial cell functioning was assessed by quantifying the activity of an apical brush border alkaline phosphatase produced by differentiated human intestinal Caco-2 using the Alkaline Phosphatase Assay kit (P0321, Beyotime). In this study, the supernatants of cells cultured for 1–5 days (50 μl) were transferred to a 96-well plate, and substrate solution was applied to the solution or standard incubation at 37°C for 30 min, then the product was quantified in a microplate reader (Multiskan Ascent, Thermo Fisher, Waltham, MA, United States) at 405 nm using culture medium as a reference. The actual amount of cleaved product was estimated based on the calibration curve of 4-Nitrophenyl phosphate disodium salt hexahydrate (pNPP).

### Colony counting

Samples were diluted 10^4^, 10^5^, 10^6^, 10^7^, and 10^8^ times respectively. 100 μl for each dilution was added into LB solid medium. For LGG, 100 μl of each dilution was added into MRS solid medium and sealed. Each dilution was repeated three times. After inverted culture for 24 h, the colonies were counted, and then the actual number of colonies was calculated.

### Statistical analysis

All results and error bars in this paper are presented as mean ± standard error (SEM). For statistical evaluation of quantified data, a one-way analysis of variance (ANOVA) and Tukey-Kramer multiple comparisons test was performed using GraphPad Prism version 7 (GraphPad Software Inc., San Diego CA, United States). Differences between groups were considered statistically significant when *p* < 0.05.

## Results and discussion

### Gut-on-a-chip microsystem design

To mimic the human intestine *in vitro*, we created a microfluidic chip (Figure 1A) that supports a long-term culture of Caco-2 cells and HUVECs. Briefly, gut-on-a-chip includes two microculture chambers, porous PC membranes, sealing channels, inlets, outlets, and microchannels. The PDMS chip’s basic design (25 mm long, 14 mm wide, and 6 mm high; this size conveniently fits onto six-well plates) incorporates two microculture chambers (2.75 mm radius, 0.3 mm high) embedded with a porous PC membrane (8 μm). Here, two microculture chambers are used to provide room for cell and medium, and the porous membrane provides a region for cell adhesion. The inlet (0.26 μm wide) is narrower than the outlet pipeline (0.52 μm wide) to avoid bubble entry or generation.

The chip is primarily round, containing a flower-shaped cylinder combination. The radius of the small cylinder is 150 μm, and the space between cylinders is 5–10 μm. The design of the flower–cylinder combination can not only be used as the support point of the PC membrane to prevent it from collapsing under the gravity of the culture medium but also can delimit the range of the culture chamber. Furthermore, the cell-free zone formed *via* blocking cells in the outer ring can be utilized as a culture medium reservoir to provide more nutrients for cells. There are five Z-shaped column combinations in the middle area to support the PC membrane and intercept cells (assist inoculation when the cells are first inoculated.) Bubbles and damage to the cells can be effectively avoided because of the rounding treatment at the bend of the Z-shaped column and different sizes of microchannels. Setting the upper and lower channels at different angles prevents cell cross-inoculation (Figure 1A).

One ever-present challenge is the method to firmly and stably embed the PC film into a chip. Here, a solution inspired by Liu and Zhang’s research (Liu et al., 2018; Tian et al., 2022) effectively solves this problem. We designed two big near-semicircular concave sealing channels and two small sealing channels on the chip periphery to hold the edge of the membrane and added liquid PDMS to seal the membrane. The big sealing channel has one middle inlet and two outlets. During the sealing process (inject liquid PDMS), atmospheric pressure is balanced by two outlets near the edge of the sealing channel. Two small sealing channels were also designed near the position of the culture chamber in the middle of the tail of the upper and lower two cell injection microchannels. The small sealing channel has different shapes, but the same principles aim to firmly fix the PC membrane-embedded by PDMS in a limited space, preventing liquid from being exchanged incorrectly between the two channels before entering the culture chamber (Figure 1B).

Because the intermediate porous membrane allows material interchange between the two channels, color mixing occurring in the chip flowthrough experiment is reasonable (Figure 1C). In some experiment (like ELISA), a relatively large volume of cell supernatant was needed, so we also designed another chip to collect enough cell supernatant. The chip is shown in Supplementary Figure S1. The manufacturing principle is similar, except that the structure of the cell culture chamber is different.

The new chip solve the problem that a thin and porous polydimethylsiloxane (PDMS) lay in the middle of the old chip (Kim et al., 2012; Kim and Ingber, 2013; Kim et al., 2016; Villenave et al., 2017; Shin et al., 2019; Ambrosini et al., 2020; Nelson et al., 2021) is difficult to manufacture. But also solves the problem that the adhesive force of the PC film and the PDMS film is not strong enough, and balances the durability and biocompatibility of the chip.

### Human intestinal model on a chip

**FIGURE 2:**
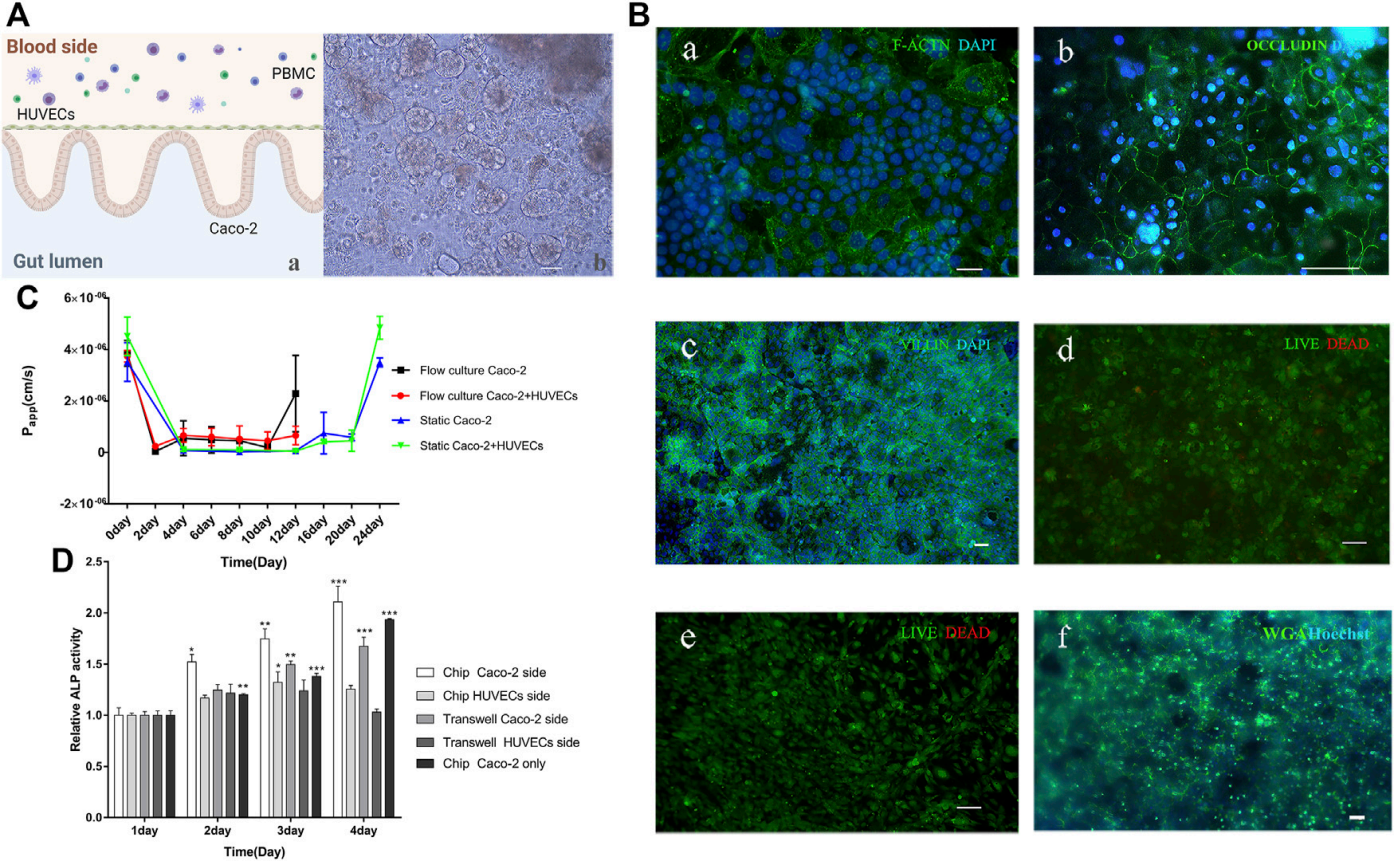
Human gut-on-a-chip. **(A)** A schematic diagram and a micrograph of the gut-on-a-chip. a: A schematic of the intestinal system; b: The bright-field images of a five-day-old gut-on-a-chip, the wavy 3D epithelial structures were observed in the microfluidic chip (Scale bar, 100 μm). **(B)** Immunofluorescence images of gut-on-a-chip culture for 5 days. a: F-actin (green); Caco-2 cell nuclei (blue); b: Occludin (green); HUVECs cell nuclei (blue); c: villi (green); Caco-2 cell nuclei (blue); d: showing the viability of the Caco-2 cells (live cells in green, dead in red); e: showing the viability of the HUVECs cells (live cells in green, dead in red); f: WGA (green); Caco-2 cell nuclei (blue), (Scale bar, 50 μm). **(C)** Apparent paracellular permeability (Papp) is measured by real-time quantitating fluorescent dextran 3-5 KDa transport through the Caco-2 monolayer or coculture with HUVECs cultured in static Transwell^®^ chamber for 24 days or in the flow culture microfluidic chip for 12 days. **(D)** Intestinal alkaline phosphatase (ALP) activity of cocultured in the static Transwell^®^ system or flow coculture in the microfluidic chip, or only Caco-2 flow culture in a chip for 1–4 days. For the coculture system, the Caco-2 and HUVECs side were measured, respectively. (chip Caco-2 side, chip HUVECs side, Transwell Caco-2 side, and Transwell HUVECs side) (*n* = 3; *p < 0.05, **p < 0.005, ***p < 0.0005).

To mimic the intestinal system, we cultured cells either in the static Transwell^®^ chamber or in the flow microfluidic chip, adding PBMC on the HUVECs side to mimic the immune system [Figure 2A (a)]. After 5 days of culture, consistent with previous studies, when gut-on-a-chip were cultured on the collagen type I hydrogel and Matrigel-coated membrane under continuous perfusion (60 μl/h), mimicking the fluid flow of the human body, the gut-on-a-chip spontaneously formed undulating villi-like structures [Figure 2A (b)]. Immunofluorescence detected F-actin expression on the lumen side, and we found that Caco-2 cells spontaneously form structures with a polarized, differentiated columnar epithelium with a tight brush border [Figure 2B (a)]. It showed a clear signal of the tight junction protein on the HUVECs cell edge, indicating that HUVECs formed a tight connection on the chip [Figure 2B (b)].

Immunofluorescence experiments proved the villi formation by detecting the villi protein expression [Figure 2B (c)]. Additionally, the Caco-2 cells and HUVECs have high viability (>99%) [Figure 2B (d, e)]. The glycocalyx in mucus was stained by WGA, which indirectly proved the formation of intestinal mucus [Figure 2B (f)]. Acidic mucopolyaccharides staining by alcian blue also proved the mucus was there (Supplementary Figure S2A). Previous researchers discovered that when cells were cultured in a Transwell^®^ chamber, it took at least 3 weeks to build intestinal barrier function (Vizoso Pinto et al., 2009; Calatayud et al., 2019), but when cells were cultured in the chip, the same effects could be achieved in only 5 days (Kim et al., 2012; Kim and Ingber, 2013; Kim et al., 2016; Jalili-Firoozinezhad et al., 2018; Nikolaev et al., 2020; Nelson et al., 2021). The intestinal mucus was present when cultured on the chip, but not in the Transwell^®^ chamber (Kim and Ingber, 2013; Jalili-Firoozinezhad et al., 2019).

The apparent permeability coefficient (*Papp*) of the gut model correlates with human intestinal absorption (Hubatsch et al., 2007). Thus, we compared the *Papp* value of Caco-2 layers or Caco-2 with HUVECs growing under static Transwell^®^ chambers or microfluidic chips with flow culture. By measuring *Papp* using fluorescent dextran with 3–5 kDa, we found that on day 12, the coculture (Caco-2 and HUVECs) model in a static Transwell^®^ chamber reached a minimum Papp (5.068 × 10^8^ cm/s), which equals to day 2 of coculture on the chip (Figure 2C). Besides, on day 20 (mature state of the gut model in a static Transwell^®^ chamber), the Papp value (4.572 × 10^7^ cm/s)of the gut model equals day 4 or day 6 of the gut model culture on the chip. Caco-2 model has a similar phenomenon. Based on the whole data analysis, it proved that gut-on-a-chip matured in about 5 days. We used dextran with different molecular weights (3–5, 40, and 70 kDa) to detect *Papp* values of two models, which were detected in gut-on-a-chip on day 4, in Transwell^®^ on day 4 and day 20. The results showed that the higher the molecular weight of dextran, the smaller the *Papp* value, and the *Papp* of the gut-on-a-chip was higher than that of Transwell^®^ on day 4, which was similar to the results of Transwell^®^ on day 20. There may be more cell stacks in the static culturing of Transwell^®^, resulting in poor system permeability (Supplementary Figure S2B). The increased permeability of intestinal cells on the chip might be due to the timely removal of dead cells by flow culture. Additionally, the coculture model keeps a stable and higher *Papp*, demonstrating that HUVECs may help the small intestine construct an intestinal barrier model, and make its barrier and absorption superior, which is consistent with previous research (Jing et al., 2020) (Figure 2C). The results demonstrated that the flow cultured gut-on-a-chip achieved a mature and stable barrier function for about 5 days.

Researchers found that intestinal alkaline phosphatase (ALP) plays a vital role in the integrity and function of the intestinal barrier structure (Nakano et al., 2009). ALP is released into the intestinal lumen *via* the apical membrane of intestinal epithelial cells (Han et al., 2001; Van Biervliet et al., 2003; Lisle). Thus, we tested the enzyme activity of ALP on both Caco-2 and HUVECs sides in three models: coculture in Transwell^®^, coculture on a chip, or only Caco-2 cultured on a chip. The results showed that the ALP activity on the coculture chip was higher than the ALP activity on only Caco-2 cells on the chip. ALP activity of the Transwell^®^ static coculture was lower than that of the flow coculture on the chip. In addition, the secretion of ALP from the Caco-2 side and HUVECs side was significantly different, and the difference increased with time, indicating the polarization degree of Caco-2 cell is increasing. The above results prove that the Caco-2 cultured for 4 days exhibited obvious ALP secretion and secretion speed faster on the chip than in the Transwell^®^ chamber (Figure 2D), and HUVECs contribute to the cytodifferentiation of Caco-2 cells. In summary, the on-chip intestinal tract we constructed formed an intestinal microstructure, expressed specific proteins, had a barrier function, produced intestinal mucus, and secreted ALP, so it could well simulate the intestinal tract *in vivo*.

To sum up, in order to simulate the physiological structure and function of the intestine to the greatest extent. The gut-on-a-chip contains the intestinal cavity, the vascular cavity. PBMCs was used to mimic the immune system, colonized probiotics are used to simulate the physiological state of the intestine. The shear forces are simulated using syringe pumps.

### Interaction between different types of bacteria and the host gut

LGG exists in the intestines of humans and animals (Kim et al., 2012). LGG becomes a biological barrier layer of the intestinal mucosa by colonizing and reproducing on host intestinal epithelial cells, improving the barrier ability of the host’s intestinal mucosa. Nevertheless, if drug-resistant bacteria colonize and reproduce uncontrollabe in the digestive system, the host’s intestinal flora will become unbalanced, prolonging disease recovery time and making treatment more difficult, which may endanger the host’s life in severe cases.

**FIGURE 3:**
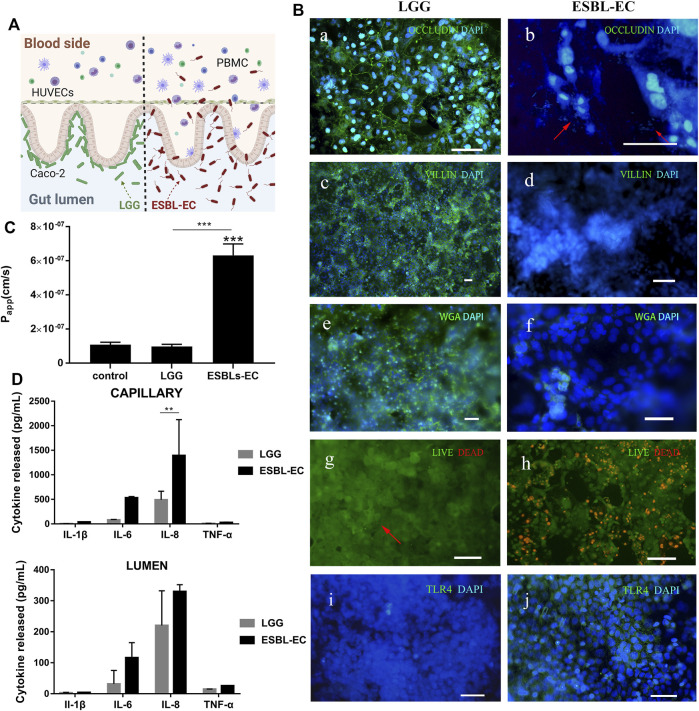
Interaction between different bacteria and host gut **(A)** A schematic of ESBL-EC or LGG coculture with gut-on-a-chip. **(B)** Immunofluorescence images. a, b: Occludin (green); HUVECs cell nuclei (blue), arrow point to ESBL-EC; c, d: villi (green); Caco-2 cell nuclei (blue); e, f: WGA (green); Caco-2 cell nuclei (blue); g, h: showing the viability of the Caco-2 cells (live cells in green, dead in red), arrow point to LGG; i, j: TLR4 (green); Caco-2 cell nuclei (blue), (Scale bar, 50 μm). **(C)** Apparent paracellular permeability (Papp) was measured by quantitating fluorescent dextran of 40 KDa transport through the Caco-2 after drug-resistant bacteria were cultured in the microfluidic chip for 1 day. **(D)** Detection of inflammatory factors IL-1β, IL-6, IL-8, and TNF-α from the lumen layer and capillary layer by ELISA (*n* = 3).

To investigate the effects of probiotics and pathogenic bacteria on the host, the probiotic LGG or intestinal drug-resistant bacteria ESBL-EC was added to the intestinal lumen side (Caco-2 side), as shown in Figure 3A. The HUVCE side of the LGG-added gut-on-a-chip remained tightly connected, but the HUVEC tight junction of the ESBL-EC-added chip was damaged [Figure 3B (a, b)], and a large amount of ESBL-EC could be seen simultaneously on the HUVECs side [see the red arrow in Figure 3B (b) and Supplementary Figure S3A]. Furthermore, compared with probiotic LGG, ESBL-EC harmed the gut microvilli structure [Figure 3B (c, d)], reduced the secretion of intestinal mucus [Figure 3B (e, f)], and dramatically enhanced cell mortality [Figure 3B (g, h)], demonstrating that ESBL-EC destroys the distinctive intestinal structure. However, there were no morphological changes in the presence or absence of LGG, demonstrating that LGG did not destroy the microstructure and function of the gut-on-a-chip. LGG was observed on the staining of intestinal cells, which gathered on intestinal epithelial cells. [see the position of the red arrow in Figure 3B (g) and Supplementary Figure S3B]. In addition, toll-like receptor 4 (TLR4) was expressed after infection with ESBL-EC but was barely expressed in the LGG-treated group [Figure 3B (i, j)]. It proves that TLR4 might recognize the lipopolysaccharide lipids on the ESBL-EC membrane and activate the inflammatory response through the NF-κB or JNK/SAPK pathways (Abreu1 and Abreu, 2007; Cario, 2005).

By investigating the effects of the two bacteria on small intestinal barrier function, we discovered that LGG did not damage the small intestinal barrier, but that adding ESBL-EC resulted in a significant increase in *Papp* (from 1 × 10^7^ to 6.5 × 10^7^ cm/s), which may be attributed to the destruction of the intestinal microstructure after ESBL-EC infection (Figure 3C). We applied ELISA kits to detect four inflammatory factors (IL-6, IL-8, IL-1β, and TNF-α) to prove the occurrence of inflammation. The inflammatory factors produced from the capillary side and secreted to the lumen side increased, proving the occurrence of inflammation (Figure 3D). This result was consistent with previous studies (Kim et al., 2016).

In summary, we demonstrated that the intestinal barrier function remains intact after coculture with LGG. ESBL-EC results in intestinal barrier destruction, microstructure damage, and the production of key proinflammatory cytokines (IL-8, IL-6, IL-1β, and TNF-α), which is consistent with previously reported results (Kim et al., 2016).

### The therapeutic effect of probiotics and antibiotics on enteritis caused by ESBL-EC

**FIGURE 4:**
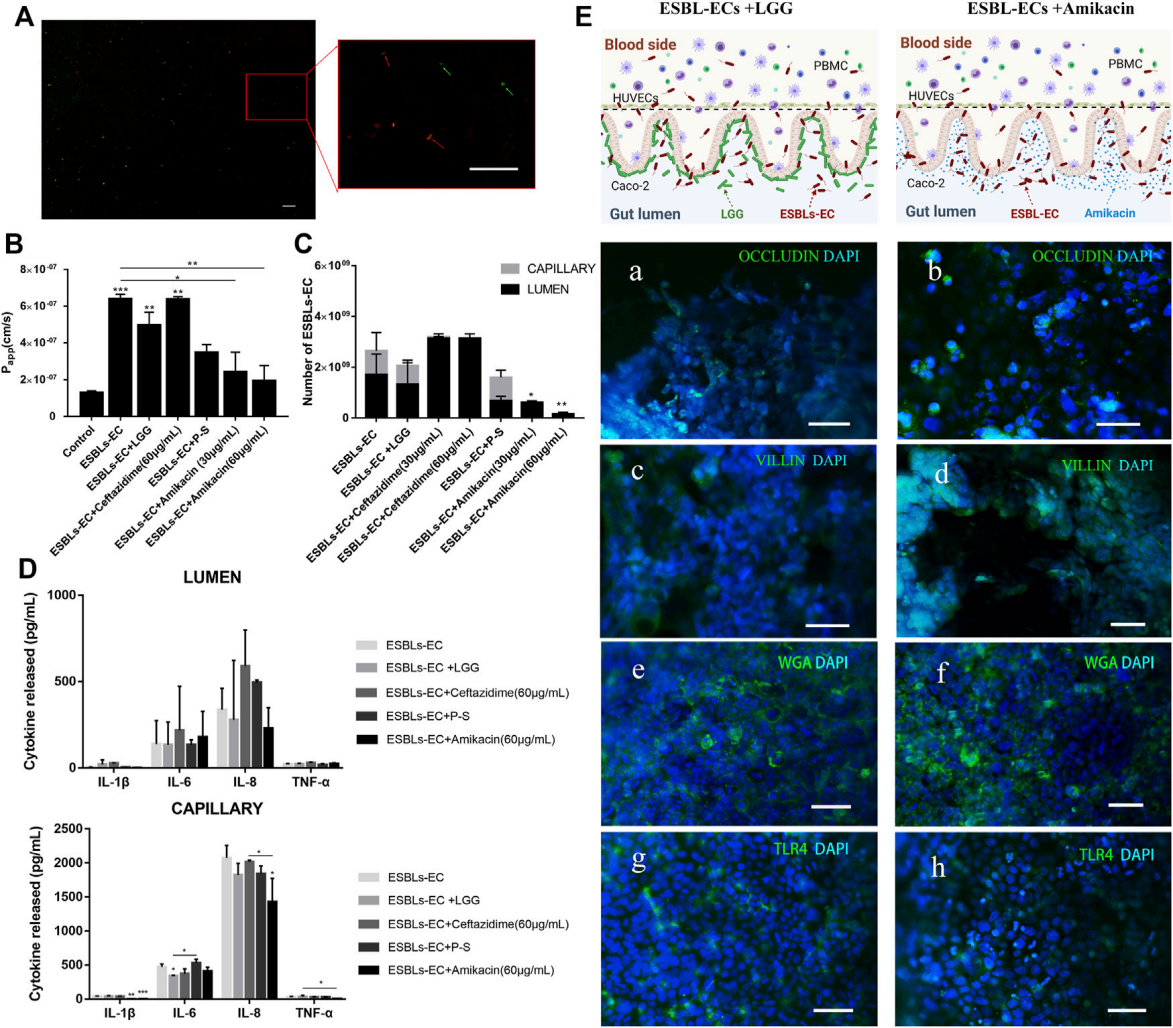
Treatment of enteritis caused by drug-resistant bacteria with probiotics and antibiotics. **(A)** Photographs of living microorganism fluorescence staining. LGG: green; ESBL-EC: red, the arrow points to microorganisms (Scale bar, 10 μm). **(B)** After being treated with different drugs in an enteritis chip, apparent paracellular permeability (Papp) was measured by quantitating fluorescent dextran of 40 KDa transport through the Caco-2. **(C)** The number of bacteria of enteritis chip after being treated with different drugs. **(D)** Detection of inflammatory factors IL-1β, IL-6, IL-8, and TNF-α from the lumen layer and capillary layer after treatment through different drugs (*n* = 3). **(E)** The schematic diagram of adding anti-inflammatory drugs (Amikacin) or LGG to the enteritis model and immunofluorescence images of enteritis chip with different treatments. a, b: Occludin (green); HUVECs cell nuclei (blue); c, d: villi (green); Caco-2 cell nuclei (blue); e, f: WGA (green); Caco-2 cell nuclei (blue); g, h: TLR4 (green); Caco-2 cell nuclei (blue), (Scale bar, 50 μm).

We used LGG and antibiotics (Ceftazidime, Amikacin, and P-S) as drugs to explore the therapeutic effects on enteritis caused by drug-resistant bacteria. LGG maintains the microecological balance in the intestinal tract of the host by regulating the structure of the intestinal community (SILVA et al., 1987; Jing et al., 2020). LGG and ESBL-EC were cocultured on the chip, and the microorganisms were subjected to live cell staining. LGG and ESBL-EC were stained green and red, respectively (Figure 4A). The red and green fluoresces could be seen clearly from the partially enlarged view on the right, and the yellow color may be due to the overlap of the two bacteria. It was, therefore, demonstrated that the microorganisms remained in good condition in the gut-on-a-chip, consistent with previous reports (Kim et al., 2012; Jalili-Firoozinezhad et al., 2019).

Next, we wonder about the effects of different drugs on the enteritis model. Both *Papp* value and bacterial counts appeared to decrease in the LGG treatment group, but no significant difference was found compared with those in the ESBL-EC group (Figures 4B,C). However, the *Papp* value of the gut-on-a-chip model after the Amikacin treatment was significantly lower than that of the ESBL-EC group, whether at high or low concentrations, whereas there was no significant difference between the other antibacterial drugs treatment groups (Figure 4B). The number of bacteria reduced significantly after Amikacin therapy but not after Ceftazidime treatment; P-S tended to inhibit the growth of resistant bacteria, but the difference was not significant. A small number of bacteria were also found on the capillary side (Figure 4C), indicating that microorganisms can reach the capillary layer through the lumen. It may be because ESBL-EC disrupts the intestinal microstructure, and a similar result may also be obtained when that vascular intercellular space is increased due to the inflammatory reaction. We found that ESBL-EC showed resistance to Ceftazidime but susceptibility to Amikacin, demonstrated by the inhibition zone diameter of 0 and 18 mm (Supplementary Figure S4), respectively. Further proves that Ceftazidime was ineffective against ESBL-EC at normal drug concentrations, whereas Amikacin was effective. The above findings revealed that Amikacin inhibits ESBL-EC reproduction, whereas P-S and LGG have poor effects; ESBL-EC showed resistance to Ceftazidime.

To detect the anti-inflammatory effect of different drugs, we evaluated the inflammatory factors of the lumen and capillary (Figure 4D). The results showed that IL-6 expression on the capillary side in the LGG treatment group was significantly lower than that in the ESBL-EC group, and IL-1β expression on the capillary side in the P-S treatment group was significantly lower than that in the ESBL-EC group. Finally, IL-1β, IL-8, and TNF-α expressions on the vascular side in the Amikacin treatment group were all significantly lower than those in the ESBL-EC group. However, there was no significant difference between the Ceftazidime treatment group and the ESBL-EC group. Because monocytes in blood vessels release inflammatory factors to the lumen, inflammatory factor was also detected on the intestine side. Inflammation is related to ESBL-EC over-growth and damage to the intestinal microstructure; TLR4 further secretes cytokines by recognizing ESBL-EC, which stimulates the recruitment of additional immune cells, thereby enhancing inflammatory response through a positive feedback loop.

The results of immunofluorescence staining of marker proteins revealed partial recovery of the tight junction between HUVECs cells [Figure 4E (a, b)], the structural integrity of microvilli [Figure 4E (c, d)], as well as intestinal mucus [Figure 4E (e, f)] in the ESBL-EC and LGG coculture group, but they did not return to control levels. TLR4 expression in the LGG group was lower than of the ESBL-EC group [Figure 4E (g, h)], indicating that inflammation may be partially suppressed. There were similar results between the Amikacin treatment group and the LGG treatment group; however, the performance of the Amikacin treatment group exceeded that of the LGG treatment group. These results indicated that both LGG and Amikacin improved the microstructure and function damage of the small intestine caused by drug-resistant bacteria, but Amikacin works better. It proved that Amikacin is effective for inflammation caused by ESBL-EC, whereas P-S and LGG have no pronounced effect, and Ceftazidime is entirely ineffective. Drug resistance test results are consistent with those from the hospital’s laboratory department.

According to “Monitoring Results of Bacterial Resistance in CHINET China (January–December 2021)” (http://www.chinets.com/Data/AntibioticDrugFast) released by the Institute of Antibiotics, Huashan Hospital Affiliated to Fudan University, *E. coli* ranked first in the clinical isolates of drug-resistant strains. From the analysis of the geographical distribution of the detection rate of important drug-resistant pathogens, it can be seen that the detection rate of drug-resistant bacteria in Shanghai ranks second in China (http://www.carss.cn/Report/Details/808).

Ceftazidime, as the third generation of semi-synthetic cephalosporins, has been widely used in clinic. The nationwide resistance rate of *E. coli* to ceftazidime was 51.6%, while the resistance rate in Shanghai was significantly higher than the national average level. Amikacin, as an aminoglycoside antibiotics, occupies a unique position in the clinical treatment of infection due to its good antibacterial activity and low price, and increasing the possible drug resistance risk. Therefore, it is of great significance to test the drug-resistant bacteria with these two commonly used antibiotics. Although only a limited number of antibiotics were used for testing and verification in this study, the gut-on-a-chip system can be used as a high-throughput unit detection module to achieve high-throughput drug resistance detection. Unlike general clinical drug resistance detection, the model is conducive to tracking the changes in cell morphology and metabolism under treatment. Besides, this model not only has the function of drug resistance detection, but also can be used for mechanism research. In the future, it is expected to use host cells for model construction, in order to achieve personalized treatment needs.

## Conclusion

Herein, we constructed a gut-on-a-chip model and explored the relationship between intestinal flora and the host. We used probiotics (LGG) and drug-resistant bacteria (ESBL-EC) to simulate intestinal flora’s normal or unbalanced state. When LGG is implanted in the intestine, the gut microstructural like microvilli and mucous, and barrier function do not receive any influence. After ESBL-EC stimulation, we found the following: 1) the microstructure was severely damaged; 2) the mucus layer disappeared; 3) the barrier function was impaired; and 4) inflammatory factor and TLR4 expression were detected, which may induce more intense inflammatory response through the NF-κB or JNK/SAPK pathways. After treatment with probiotics (LGG) and different antibiotics for drug-resistant bacteria, we found that LGG and P-S have a limited effect on reducing damage to the gut microstructure and the function, as well as an inflammatory response caused by ESBL-EC. The antibiotic Amikacin effectively inhibits inflammation and protects the structure and function of the small intestine from destruction, but ESBL-EC is insensitive to antibiotic Ceftazidime. These findings are consistent with clinical test results.

Above all, we constructed a gut-on-a-chip system, which used a new chip with more solid membrane-embedded technology. We added human peripheral blood mononuclear cells to mimic the immune system and used pumps to mimic the shear force *in vivo*. Drug-resistant bacteria were introduced into the gut-on-a-chip to explore the effect of drug-resistant bacteria on the intestine. This technology can simulate the occurrence of enteritis and evaluate different treatment regimens. Biomimetic chips can also evaluate drug side effects and cell culture modules for high-throughput drug screening. Nevertheless, because of the limitations of the laboratory platform, gut biopsy tissue is not used to construct the gut-on-a-chip. However, this does not affect the great potential development prospects of this technology in the future.

## Data Availability

The original contributions presented in the study are included in the article/Supplementary Material, further inquiries can be directed to the corresponding authors.
