# Supplementary material for: Primary exploration of host–microorganism interaction and enteritis treatment with an embedded membrane microfluidic chip of the human intestinal–vascular microsystem
Source: Front Bioeng Biotechnol. 2022 Dec 6;10:1035647. doi: 10.3389/fbioe.2022.1035647 (PMC9763581; doi:10.3389/fbioe.2022.1035647)
Supplement: Supplementary file 1 [file DataSheet1.pdf]

Primary exploration of host–microorganism interaction and enteritis treatment with an embedded membrane microfluidic chip of the human intestinal–vascular microsystem

Authors: Wei Zhao [1], Yuhan Yao [1], Tong Zhang [1], Huijun Lu [1], Xinlian Zhang [1], Linlin Zhao[2], Xi Chen[1], Jinhui Zhu[1], Guodong Sui,[1]*and Wang Zhao, [1]*

[1] Shanghai Key laboratory of Atmospheric Particle Pollution Prevention (LAP3), Department of Environmental Science & Engineering, Fudan University, Shanghai, China

[2] Shanghai Changhai Hospital Department of Gastroenterology, Shanghai, China

Corresponding Authors

* E-mail: gsui@fudan.edu.cn

* E-mail: zhaowang@fudan.edu.cn

Keywords: gut-on-a-chip; embedded membrane chip; ESBL; enteritis; treatment;

Supporting Information

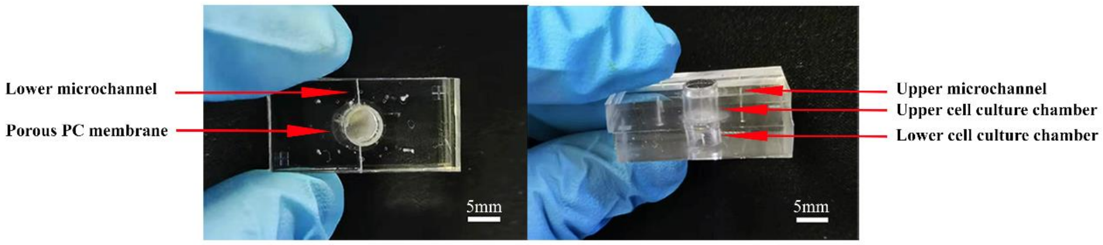

Figure S1: Microsystem for gut-on-a-chip. Photographs of the chip's physical diagram were taken from various angles. The PC membrane was inserted into the middle of the two cell culture chambers using the sealing layer. Two culture chambers are positioned on the PC membrane's upper and lower rooms with a larger volume, which is suitable for the detecting inflammatory factors.

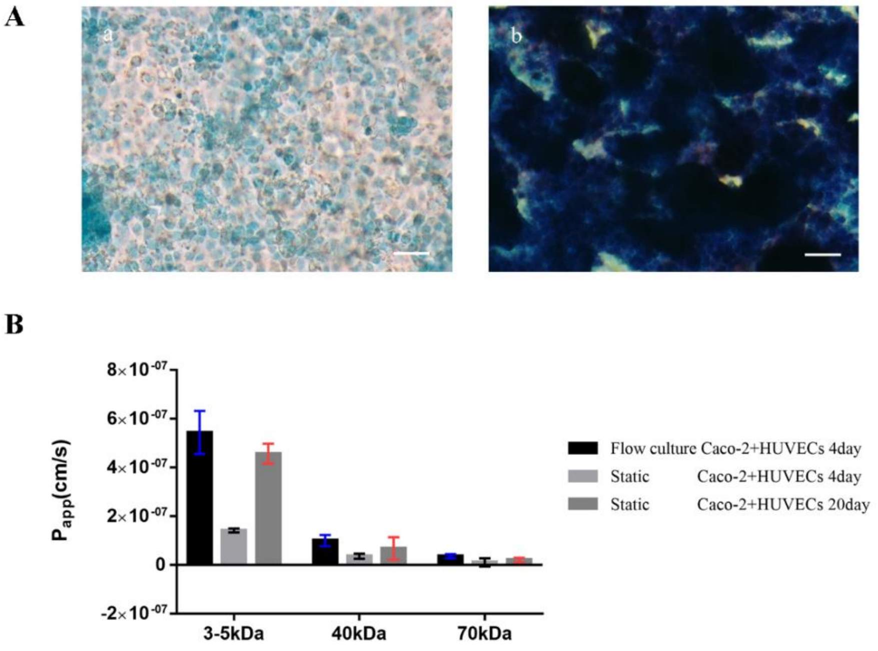

Fig S2:Human gut-on-a-chip. (A). Images of intestinal mucus acidic mucopolyaccharides staining. a: Caco-2 seed in Transwell culture for 21 days; b:gut-on-a-chip flow culture for 5 days. (B). Detection of apparent permeability coefficients (*Papp*): The *Papp* value for the small intestinal model co-cultured with different equipment (Flow culture represents gut-on-a-chip; static represents co-culture in the Transwell® chamber) was measured by quantitating fluorescent dextran 3-5kDa,40kDa and 70kDa at day 4 and/or day 20. Scale bar, 50μm.

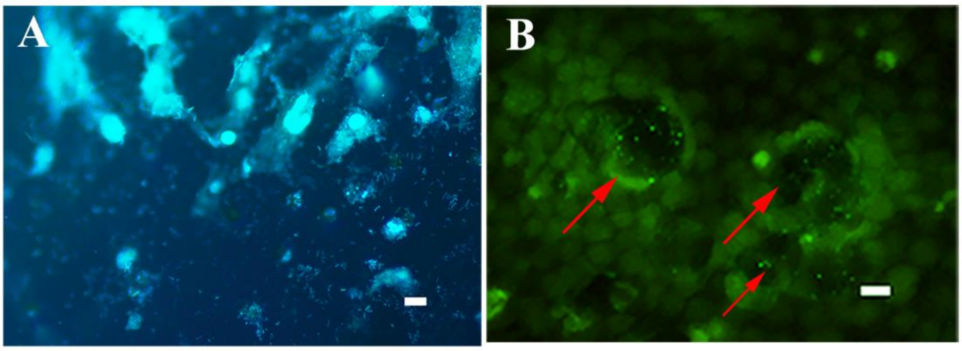

Fig S3 LGG and ESBL-EC staining. A: ESBL-EC staining: ESBL-EC was injected into the intestinal lumen of the chip. When HUVECs were stained with DAPI, ESBL-EC was also stained and observed inadvertently (Scale bar, 10μm). **B: LGG staining:** LGG were stained by CMFDA, cultured

and observed after injection into the intestinal cavity of the mature gut-on-a-chip, and photographed under a fluorescence microscope. The red arrow pointed to LGG.

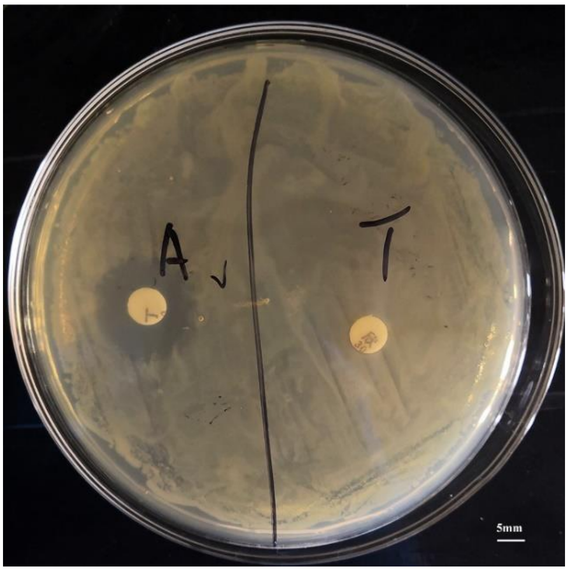

Fig S4: Antimicrobial susceptibility test: Two kinds of antibiotic paper (30μg/ piece) were spread on the plate coated with ESBL-EC. The letters A and T stand for amikacin and ceftazidime, respectively.

Additional proof

Fig.2B(b)

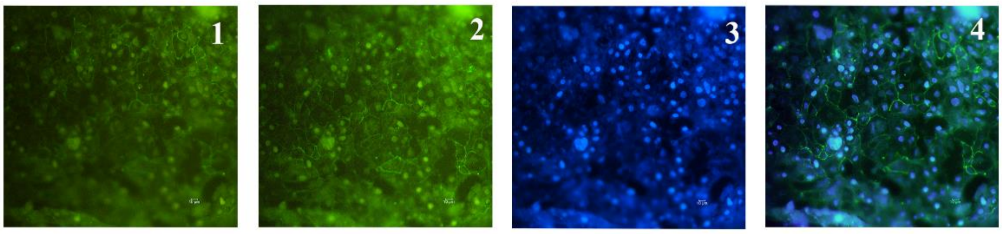

Fig.3B(b)

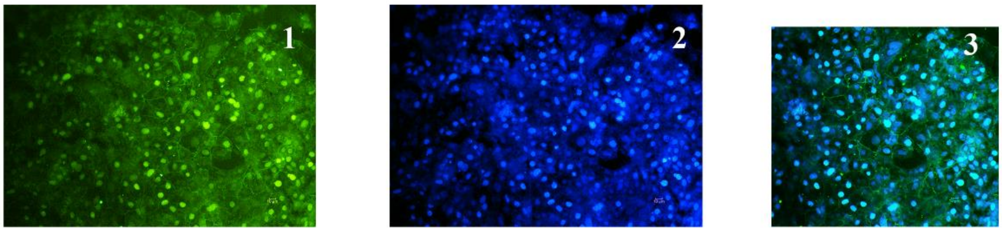

Fig2B(b) **(1)** Green represents the Occludin of HUVECs, and this picture is focused on the top part of the picture. **(2)** Green represents the Occludin of HUVECs, and this picture is focused on the lower and middle part of the picture. **(3)** HUVECs cell nuclei were stained by DAPI(blue). **(4)**The merge image of 1,2 and3. (Scale bar, 10μm).

Fig3B(b) **(1)** Green represents the Occludin of HUVECs. **(2)** HUVECs cell nuclei were stained by DAPI(blue). **(3)** The merge image of 1and 2. (Scale bar, 10μm).

In figure 2B(b). (1) and (2) represent tight connection of HUVECs in different focal planes. (1) is focuse on that top part of the picture. (2) is focused on the middle and lower part of the picture. (3) represents the cell nuclei of HUVECs. In this set of graphs, it can be seen that tight junction protein of HUVECs is expressed as reticular junction, i.e. tight junction. Figure 3 B(b) is the same, and the original uncut picture is kept for the convenience of observation. Thus, it can be demonstrated that HUVECs remain tightly connection on the chip with or without LGG colonization.
